# Using video-reflexive ethnography to capture the complexity of leadership enactment in the healthcare workplace

**DOI:** 10.1007/s10459-016-9744-z

**Published:** 2016-12-30

**Authors:** Lisi Gordon, Charlotte Rees, Jean Ker, Jennifer Cleland

**Affiliations:** 1 School of Management, University of St Andrews, The Gateway, North Haugh, St Andrews, KY16 9RJ Scotland, UK; 2 Faculty of Medicine, Nursing and Health Sciences, Monash University, Melbourne, VIC Australia; 3 NHS Education for Scotland, East Region Ninewells Hospital, Dundee, Scotland, UK; 4 Institute of Education in Medical and Dental Sciences, University of Aberdeen, Aberdeen, UK

**Keywords:** Leadership, Leadership education, Video-reflexive ethnography, Interprofessional, complexity theory

## Abstract

Current theoretical thinking asserts that leadership should be distributed across many levels of healthcare organisations to improve the patient experience and staff morale. However, much healthcare leadership education focusses on the training and competence of individuals and little attention is paid to the interprofessional workplace and how its inherent complexities might contribute to the emergence of leadership. Underpinned by complexity theory, this research aimed to explore how interprofessional healthcare teams enact leadership at a micro-level through influential acts of organising. A whole (interprofessional) team workplace-based study utilising video-reflexive ethnography occurred in two UK clinical sites. Thematic framework analyses of the video data (video-observation and video-reflexivity sessions) were undertaken, followed by in-depth analyses of human–human and human–material interactions. Data analysis revealed a complex interprofessional environment where leadership is a dynamic process, negotiated and renegotiated in various ways throughout interactions (both formal and informal). Being able to “see” themselves at work gave participants the opportunity to discuss and analyse their everyday leadership practices and challenge some of their sometimes deeply entrenched values, beliefs, practices and assumptions about healthcare leadership. These study findings therefore indicate a need to redefine the way that medical and healthcare educators facilitate leadership development and argue for new approaches to research which shifts the focus from *leaders* to *leadership*.

## Introduction

Regulatory bodies endorse the importance of incorporating leadership education at all stages of healthcare professionals’ careers (Garling 2008; McKimm and O’Sullivan 2011; Frances 2013). Furthermore, the literature identifies the need for education programmes focussing on the development of personal and interpersonal competencies linked to good healthcare leadership (Calhoun et al. 2008; Swanwick and McKimm 2012; Gabel 2014). Unfortunately, evaluations of healthcare leadership education interventions indicate, at best, only modest effects and that more robust research is necessary, particularly in terms of better understandings of healthcare leadership in context and what implications this may have for educational practice (Steinart et al. 2012; Straus et al. 2013). Moreover, much research has lacked consideration of current theoretical discourses of healthcare leadership, making it difficult to generalise conceptually across contexts (Gordon et al. 2015). In this paper, we join the discussion by exploring healthcare leadership in context. We argue, through our research, the need to rethink how we approach healthcare leadership education. We do this by presenting the findings from a video-reflexive ethnography study underpinned by complexity theory that explored leadership enactment in the healthcare workplace.

### Healthcare leadership and complexity theory

There is growing opinion that traditional individualistic understandings of leadership are no longer sufficient to explain successful leadership (Marion and Uhl-Bien 2001; Plowmand and Duchon 2008; Uhl-Bien and Ospina 2012). Current thinking asserts that leadership should be distributed throughout a healthcare organisation in order to learn how to resolve problems as they arise, a potentially unpopular notion that contradicts the widely held view that leaders are the problem-solvers at the top (Martin 2011). These distributed leadership practices are seen to contribute to an improved patient experience; reduced errors, infection and mortality; increased staff morale and reduced staff absenteeism and stress (West et al. 2015).

Complexity theory offers an alternative approach to the study of leadership and leadership education within healthcare. The concepts of interconnectedness and non-linearity associated with complexity thinking are recognised as ‘normal operating conditions’ within healthcare organisations (Pslek and Greenhalgh 2001; Kernick 2002; Weberg 2012). Complexity leadership theory responds to a modern changeable healthcare environment in which new learning and new work patterns are often required (Uhl-bien et al. 2008). This is dissimilar from technical issues that are resolved through current knowledge (for example, a new electronic patient record) and experiences possessed by individuals (Parks 2005). As such, healthcare leadership becomes an “emergent interactive dynamic” (Uhl-Bien et al. 2008: p. 187).

There are several premises for complexity leadership theory: first is the assumption that leadership is co-constructed through interaction between individual healthcare professionals and healthcare teams working within complex adaptive systems (CASs) and thus it becomes an *emergent* phenomenon (Lichtenstein and Plowman 2009; Bleakley 2010). Second, a complexity leadership perspective necessitates a distinction between *leadership* and *leaders* (Uhl-Bien et al. 2008). To clarify, leaders are typically thought of as individuals who hold designated leader roles, whereas leadership is often thought of as a process involving multiple agents including those who might enact leadership and those who might enact followership according to context (Gordon et al. 2015; Lichtenstein and Plowman 2009). Third, a complexity perspective separates leadership from designated position meaning that leadership sits outside formal leadership positions and throughout the healthcare organisation (Uhl-Bien et al. 2008). Arguably as a result, leadership education should be focussed on circumstances in which healthcare teams have to learn their way out of unpredictable situations, achieving this through ‘distributed intelligence’ rather than reliance on the few that hold formal leadership positions (Parks 2005; McKelvey 2008).

Prevailing organisational researchers seek to reduce and simplify the complex environments they study focussing on top-down leadership (Hosking 1988; Kernick 2006). From a complexity theory perspective, however, focussing only on hierarchical positions within organisations does not sufficiently address the leadership process (Rost 2001; Bedian and Hunt 2006). Rather, researching leadership should instead examine the “dynamic (changing, interactive and temporal) informal interactive patterns that exist in and among organisational systems” (Uhl-Bien et al. 2008: p. 214).

Using complexity as a theoretical lens, we can pay attention to the way in which leadership emerges across multiple levels of a healthcare organisation and within multiple timescales (Lichtenstein and Plowman 2009). For example, leadership can occur: (1) in the micro-level minute-by-minute interactions of healthcare teams working together in a focussed way; (2) at a ‘meso-level’ through the daily and weekly changes in relationships and ways of working between different teams across an organisation; and (3) at a ‘macro-level’, with change occurring over weeks and months through major organisational events and emergent learning (Lichtenstein and Plowman 2009). While we discuss the influence of the meso- and macro-levels, this paper focusses primarily on micro-level leadership interactions.

### Micro-level leadership interactions: Influential Acts of Organising

Exploring leadership at a micro-level means focusing on interactions between people and what leadership means to those involved (Alvesson and Karreman 2000; Fairhurst and Uhl-Bien 2012). Key to this is that researchers “suspend the assumption of assigned leader roles to look for influential acts of organisation in the sequential flow of action [in context] by any leadership actor” (Fairhurst and Uhl-Bien 2012: p. 1045). These influential acts of organising (IAOs) are seen as moments where an individual will influence another (or others) more than they are influenced by them (Hosking 1988). Thus, a “leadership act” is enacted by “those who achieve the most influence in the course of [a] negotiation” (Hosking 1988: p. 153). Aligned with complexity thinking, seeing leadership at a micro-level in this way separates the process from designated organisational role and makes it available to all (Hosking 1988). Additionally, this viewpoint makes it possible for there to be more than one individual undertaking ‘leadership acts’ throughout the course of an interaction (Hosking 1988). Consequently, there is an argument for research that focuses on micro-leadership processes through exploration of leadership practices, leadership enactment and emergence, the embodiment of leadership and the materiality (i.e. the physical state such as paperwork or medical artefacts) of leadership (Denis et al. 2010). In healthcare education, therefore, it can be suggested that collecting interactional data through direct observation may provide opportunities to explore context and the interactional processes that are key to leadership processes (Fairhurst and Uhl-Bien 2012).

Some recent research has focussed on observing micro-leadership processes (Linguard et al. 2012; Chriem et al. 2013). Both studies collected observational and interview data from interprofessional healthcare teams in Canada, seeking to understand: the role of physician leadership within collaborative healthcare practices (Linguard et al. 2012), and how leadership practices are undertaken across boundaries in interprofessional teams (Chriem et al. 2013). The first study found that despite an articulated desire for a shared leadership approach within interprofessional teams, they observed behaviours and systems that perpetuated traditional leadership hierarchies (Linguard et al. 2012). The second study found that boundary work was central to leadership practices in various forms—both internally and externally—and embedded in a wider macro-environment (Chriem et al. 2013). It can be argued that there is on-going need to discuss the tensions between the nature of workplace leadership and the wider organisational structures and processes in which leadership is embedded. Arguably, there is a lack of literature in which the ‘everyday’ healthcare workplace environment is explored in detail and placed in the context of wider organisational systems. Furthermore, whilst the two studies discussed above shed light onto micro-level leadership interactions, they do so at the expense of detailed interactional processes that video-data would have afforded. Therefore, we present a video-reflexive ethnography (VRE) study underpinned by complexity theory, which asks how leadership is enacted (at a micro-level) in the interprofessional healthcare workplace, and what the implications of this enactment are for healthcare leadership education.

## Methods

### Study design

As discussed above, a key assumption of complexity theory is that leadership is co-constructed within interaction; communication is therefore a key component (Fairhurst and Uhl-Bien 2012). These interactions are conceptualised as dynamic and changeable over time (Uhl-Bien and Ospina 2012). Thus, our research is epistemologically grounded in social constructionism, which asserts that “all knowledge and therefore all meaningful reality as such is contingent upon human practices, being constructed in and out of interaction between human beings and their world…” (Crotty 1998: p. 42). Social constructionism is about how those “meanings” are created through relationships (Gergen and Wortham 2001). Thus, we believe that multiple, complex realities about leadership are constructed through social interactions, in both language and non-verbal interactions and our chosen methodology reflects this.

### Methodology

Visual methodologies are gaining in recognition at the interfaces between social sciences and health services research through their ability to capture the complexities of healthcare practice at the level of workplace interaction (Rees 2010; Iedema et al. 2013). Increasingly within healthcare, video is being utilised by interprofessional teams in collaboration with patients and researchers as a tool for inquiry, learning and service development (Carroll 2009). Video-reflexive ethnography (VRE) refers to a methodology that uses video, is ‘ethnographic’ in that the video captures participants in their ‘natural’ working environment and is ‘reflexive’ in that it involves participants exploring as a group what was captured on the video footage (Iedema et al. 2013). Central to VRE are issues of context, actions and social interactions, making visible how participants respond to each other’s behaviours and actions, and providing opportunities for repeated scrutiny of those actions and interactions (Heath et al. 2007). Thus VRE has the potential to make everyday practices visible to researchers and participants as it captures the delicate relationships between verbal and non-verbal practices (Iedema et al. 2013). VRE responds to the need to reflect the complexities and fluidity of relationships and interactions within the healthcare environment; and can reveal the “unseen” habits of everyday work (Iedema et al. 2013).

### Recruitment methods

Following ethical approval, two sites within the UK healthcare workplace were recruited; one GP Practice (Site A) and one hospital ward (Site B). Local Deaneries (who are responsible for local provision of postgraduate medical education) provided details of potential sites and key contacts were approached by email and face-to-face discussion. Site A is a medium-sized community general practice providing primary care services for a UK town and rural district. Site B is a 30-bed elderly rehabilitation ward in a district general hospital in a small UK city.

All members of the interdisciplinary teams working directly in both sites were invited to participate. Recruitment occurred face-to-face and all potential participants were approached on an individual basis and given a ‘cooling off’ period to consider their possible participation. Multi-level written consent was sought by the lead author for: observation in the workplace; observation in the workplace using video; use of video in the reflexivity sessions; participation in the video-reflexivity sessions; and use of video-recordings for educational and dissemination purposes. Most consents were obtained at this point. However, some were obtained during later stages as other staff members became involved in the study serendipitously. Table 1 presents the participant characteristics.

**Table 1:** Participant characteristics

Participant characteristics	Site A (n = 39)	Site B (n = 42)
Gender		
Male	8	7
Female	31	35
Ethnicity		
White	39	40
Non-white	0	1
Not answered	0	1
Professional role		
Doctor	11	5
Medical student	2	3
Nursing staff	13	21
Administrative staff	10	2
Allied health professional	3	8
Social worker	0	3

### Data collection

The primary author (an ex-physiotherapist) undertook data collection for this study. Whilst an outsider to the workplaces studied, her previous clinical experiences afforded some ‘insider’ knowledge, which facilitated her comfort within both clinical settings and allowed her to ‘understand’ the ‘language’ of healthcare practices and discuss aspects of working practices with participants using this language (Burns et al. 2012). Additionally, her prolonged engagement with the workplaces studied meant that a level of trust was built up between the researcher and research participants over time, thus facilitating the data collection process.

Data collection occurred in three stages: (1) a period of familiarisation and observation within the workplace; (2) video-footage was recorded of real workplace practice (video-observation stage); and (3) this footage was then edited and the edited footage played back to the interprofessional team, providing them with the opportunity to reflect on and discuss their practices (video-reflexivity stage). Note that only staff-to-staff interactions were observed and recorded.

#### Familiarisation and observation without video

Familiarisation of the researcher to the environment and the environment to the researcher allowed for the identification of possible points in the working week where video-observation could best take place (i.e. contexts where leadership interactions occurred within interprofessional teams without patient contact). Familiarisation was achieved through informal discussion with participants at various points during the working day alongside basic field notes discussed later with the co-authors. During familiarisation, the primary author attended each site for short periods (30–180 min) during normal working hours (8–6 pm) in site A on five different occasions and in site B on seven different occasions (total time for familiarisation = 19 h 50 min).

The observation without video stage involved watching practices that had been identified by the participants within the familiarisation stage as points of interprofessional interaction (with non-patient contact). In site A, the primary author was onsite for periods of between 2 and 5 h on four occasions (a total of 15 h 25 min observation time); in site B the primary author was onsite for periods of 1–4 h on four occasions (a total of 7 h 55 min observation time). During this time, basic field notes were taken which provided outline details pertaining to working practices, notes from one-to-one conversations with various participants and more detailed descriptions of the format of more formal settings, for example, multidisciplinary team meetings.

#### Video-observation

The focus of the video-observation stage was to capture leadership interactions as they occurred within the context of the interprofessional workplace. A small ‘handi-cam’ with a wide-angled lens was used. The initial intention was to video using a ‘fly-on-the-wall’ style and the majority of footage was captured in this way. However, in some footage participants would interact with the researcher behind the camera discussing their thoughts and practices whilst being videoed, with this style of videoing becoming ‘expert-apprentice’ (i.e. with participants as ‘experts’ explaining to the ‘apprentice’ researcher what they were doing and thinking). In total, 12 h, 37 min of video-observational data were collected. Table 2 provides detail of the video-observation footage captured in this stage.

**Table 2:** Details of video-observation sessions

Context	Length hr:min:sec	Summary of participants	Outline summary of footage content
Site A			
1. Communication meeting	00:56:02	All practice members present: GPs; GP trainees; practice manager; district nurses; practice nurses; community hospital nurses; health visitors; cancer nurse; practice pharmacist; medical students	Weekly meeting to discuss the management of complex patients in the practice, community and community hospital. Chaired by the practice manager
2. Diabetic meeting	00:27:24	GP and practice nurse	Weekly meeting to discuss management of diabetic patients in the practice
3. Educational meeting	02:32:19	Various GPs; GP trainees; pharmacist; practice nurses	Weekly educational meeting led by a member of staff, covering various subjects
4. Community hospital	00:11:25	GPs; community hospital nursing staff	Unplanned interactions at community hospital
5. Practice manager shadowing	00:08:14	Practice manager; nursing staff	Footage collected as part of shadowing practice manager in her office
6. Reception area	00:15:57	Reception staff; GPs; nursing staff	Footage of unplanned interactions from time spent in GP reception
7. Trainee shadowing	02:52:17	GP trainees; GPs	Footage from shadowing the 2 GP trainees in the practice
Total Site A video time	07:23:43		
Site B			
8. Board round	00:44:00	Both ward consultants; ward nursing staff; social worker; physiotherapists; occupational therapists	Twice-weekly meeting (Monday and Thursday morning) to discuss discharge plans for ward patients. Chaired by nursing staff
9. Multidisciplinary meeting	02:38:56	Nursing staff; medical consultants; social worker; occupational therapist; physiotherapists; nursing students; medical students	Weekly multidisciplinary meeting to discuss all patients’ progress and planning
10. Ward round	01:15:37	Medical consultants; foundation trainees; medical students; various nursing staff	Twice weekly consultant’s ward rounds in which medical issues discussed and patients seen
11. Informal ward-based interactions	00:36:29	All consented staff	A range of informal interactions captured outside formal activities listed above
Total Site B video time	05:14:32		

#### Video-reflexive ethnography (VRE) groups

The purpose of the video-reflexivity stage was to provide participants with the opportunity to reflect on leadership practices and discuss these as they saw them happening. The first step in this process was to edit the video-observational footage collected into short clips that could be discussed within the reflexivity sessions. Decisions about what to edit were made as a research team who met regularly during this phase to discuss the footage and how it might be edited. Editing decisions were based on several factors including: (1) range of participants and their roles; (2) researcher field notes; and (3) the researchers’ knowledge of the literature and healthcare leadership roles (Gordon 2015). Thus, the video-observational footage was edited to include what was perceived to be examples of influential acts of organising (IAOs), that is, moments where an individual had more influence than others on the course of the ensuing action (Hosking 1988).

These edited clips were then played within VRE group sessions to which all participants were invited. Within these sessions the edited clips (pertaining to that site only) were played and the lead author facilitated discussion focussing on leadership. Each VRE group began with an introduction to the session and explanation of the layout. Following this, the researcher would show an edited clip, then provide participants with the opportunity to discuss the clip generally, in terms of leadership and followership and any other aspects they particularly wanted to discuss in relation to the clips. The researcher’s role within these sessions was to facilitate the discussion through prompt questions, to ensure the discussion stayed on topic (i.e. leadership) and probe some issues in more depth. Each session was videoed using the hand-held camera on a tripod, with wide-angled lens. Unlike the video observation footage, the researcher was visible within the video-reflexivity footage. Of the raw video-data outlined in Table 2, we edited footage to 23 min for Site A and 20 min for Site B. We ran five VRE groups in Site A and seven in Site B (note that the length of each session was determined on participant availability/time); ranging from 10 to 73 min (totalling 676 min; average duration 56 min). See Table 3 for details of each VRE group.

**Table 3:** Details of VRE groups

VRE group	Length	Attendees	Clips shown
Site A			
VRE Group 1	00:59:10	District nurse x2; health visitor x2; GP trainee (ST1); practice pharmacist; practice manager; medical student; GPs x5	Communication meeting clips 1–4 Interprofessional interactions (admin) clips 1–3
VRE Group 2	00:10:44	Receptionist; assistant practice manager	Interprofessional interactions (admin) clips 1–3
VRE Group 3	00:46:51	GPs x3; practice pharmacist; practice nurse	Interprofessional interactions clip 4 (diabetes meeting) Educational meetings clips 1–3
VRE Group 4	01:08:37	GPs x2; GP trainees x2 (ST1 and ST3)	Educational meeting 3 Trainee tutorial GPST1 and GPST3 shadowing
VRE Group 5	01:01:29	District nurse; GP; Community hospital senior charge nurse; GP trainee (ST3)	Communication meeting clips 1–4 Community hospital patient
Site B			
VRE Group 6	01:06:34	Ward occupational therapist (OT); physiotherapist	All site B clips
VRE Group 7	00:36:32	Ward staff nurses x2; social worker	Board round clips 1–3 Informal Interaction clips 1–13 MDT meeting clips patients A–E
VRE Group 8	01:14:03	Ward consultants x2; physiotherapist	All site B clips
VRE Group 9	01:14:31	Medical specialty trainee (ST6)	All site B clips
VRE Group 10	00:53:21	Foundation trainee	All site B clips
VRE Group 11	01:02:07	Mental health nurse; mental health OT	All site B clips
VRE Group 12	01:01:34	Ward charge nurses x2	All site B clips

### Data analysis

A team-based approach to data analysis was undertaken whereby each author watched a selection of edited video clips and VRE group data independently. Following this, the research team met to discuss overarching themes. A coding framework was developed, which formed the basis for broad analysis of the video-observational and VRE group footage and detailed analysis of the edited video clips. In order to assist with managing the large volumes of visual data, computer assisted qualitative data analysis software (CAQDAS) was used (Lewins 2008). For these purposes, Atlas.ti (Version 7, GmbH: Berlin) was used which allowed coding (using the developed coding framework) directly onto the video footage, and to organise, interrogate and retrieve segments of data to assist with data analysis.

Across the raw video-observational footage, we identified points at which the process of leadership occurred in context (i.e. an IAO: Hosking 1988). Within these IAOs, we identified who was involved and the type of activity taking place at the time. We also identified the interactional processes occurring as part of leadership practices.

To add a further layer of analysis onto the edited video clips, we undertook a form of little ‘d’ discourse analysis. This meant paying close attention to the language-in-use in social interaction between the interprofessional healthcare teams within the edited video footage (Alvesson and Karreman 2000; Fairhurst and Uhl-Bien 2012). Detailed transcription of the edited clips, as well as repeated viewing of the video footage, allowed us to identify how language-in-interaction was used to negotiate leadership processes, for example, through the use of pronouns, questions and answers, name use, directives, pauses and hedges within talk (Rees and Monrouxe 2010; Rees et al. 2013). To augment this, and take this analysis beyond the analysis of talk alone, we also undertook visual analysis of non-verbal human–human interactions (for example, body language, physical positioning and eye contact) and human–material interactions (for example, use and/or control of artefacts: Fenwick and Nimmo 2015).

To begin to analyse the VRE groups we used ‘phasal codes’, which pay attention to the rhythm of the discussion (O’Halloran 2011). Within the VRE groups talk moved through different ‘phases’ in two different ways: (1) discussion related to the video or discussion not related to the video; and (2) discussion related to leadership or discussion unrelated to leadership. Coding the different phases allowed linkage of the discussion within the reflexivity session to our own detailed analysis of the edited clips.

## Findings

**Fig. 1:**
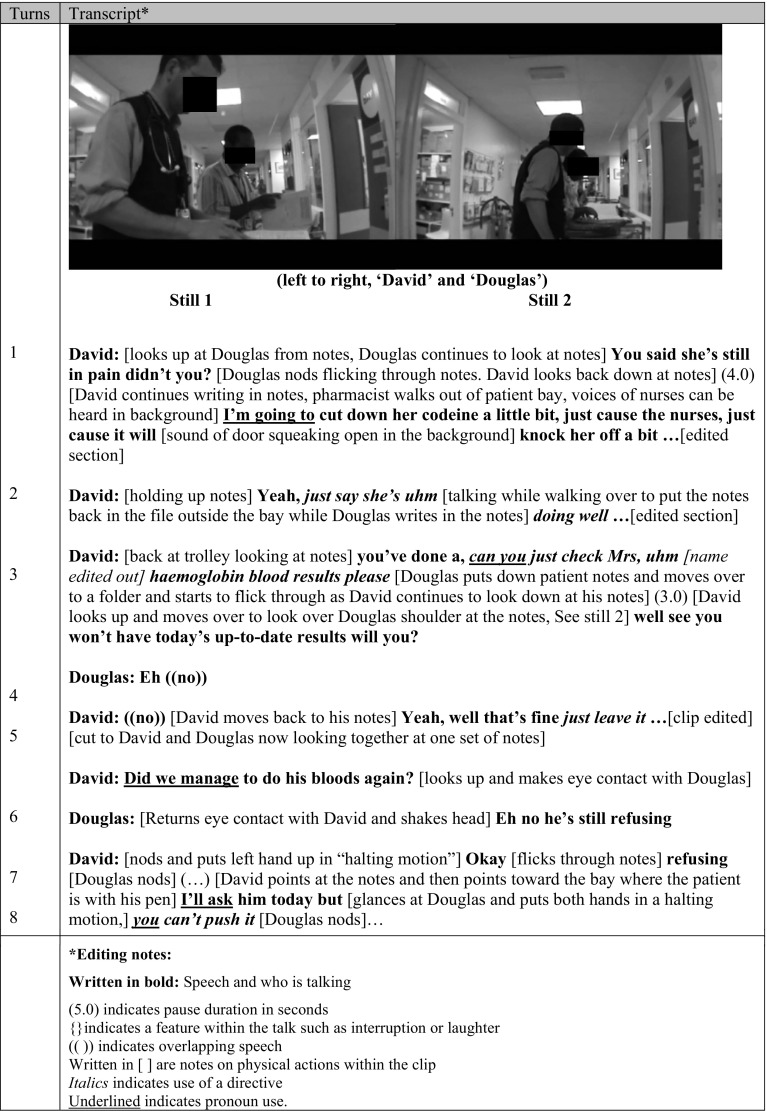
Clip taken from observational session number 10 (Ward round: shown in VREGs 6–12)

**Fig. 2:**
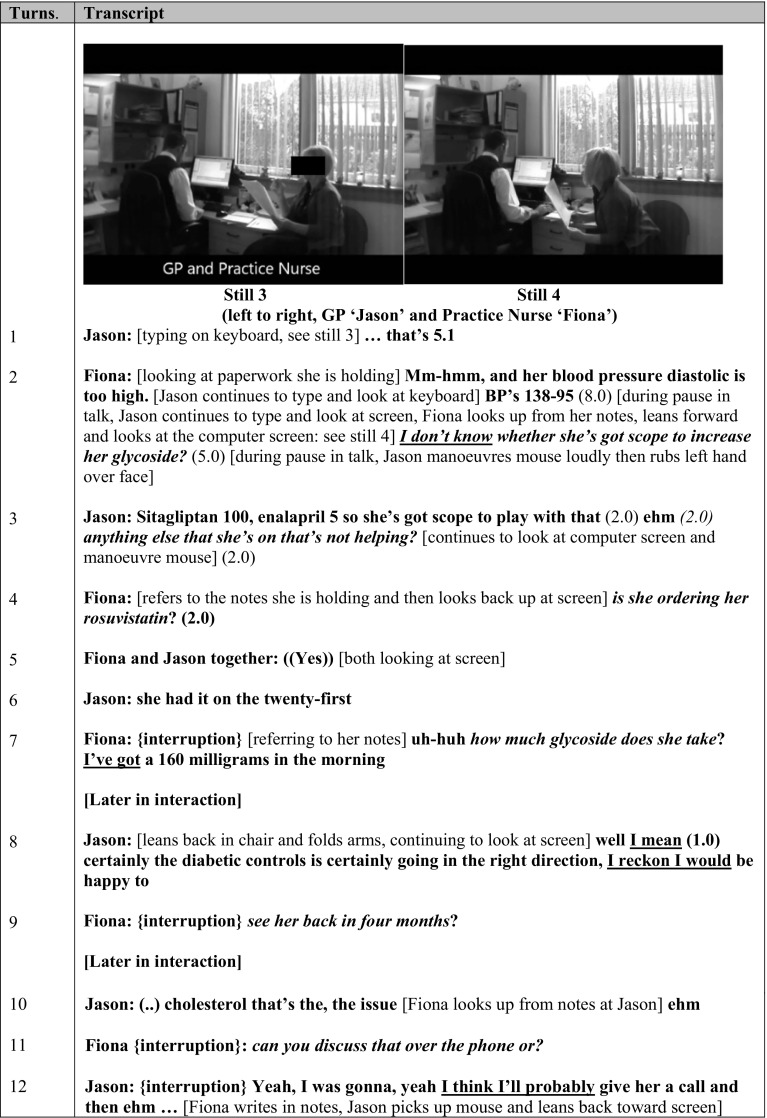
Clip from observation session 2 (Diabetic meeting: shown in VREGs 1, 3 and 5)

Within this section we present our findings in the following way: first, we focus on the processes of leadership we observed in context; second, we take a closer view at the interactional processes involved; and finally, we present what was said in the VRE groups. To illustrate our findings, we have selected two data excerpts depicting leadership interactions. These excerpts were chosen as they highlight two typical but different types of leadership interaction across our data set. We present details of the video observation footage (shown later in Figs. 1, 2) and VRE group discussion about these two interactions (shown later in Table 4). Figure 1 depicts a leadership interaction between a ward consultant ‘David’ and a foundation trainee ‘Douglas’ (both pseudonyms), recorded during the weekly consultant ward rounds in Site B (see Table 2, row 10). Figure 2 depicts a leadership interaction between a GP ‘Jason’ and practice nurse ‘Fiona’ (both pseudonyms) during a weekly diabetes meeting in Site B (see Table 2, row 2).

**Table 4:** Quotes from VRE groups (VREG)

a	VREG8	***David*** *: “… I would say with [Douglas] there’s a pretty unambiguous relationship [nods] … something that’s evolved in medicine is that, I probably knew more about the patients than him, whereas years ago he would be telling me about the patients, it’s just a fact the way medical training’s evolved … this sounds bad but they don’t really have anything useful to tell us, that sounds awful … it’s very much me directing him … they’re rotating so much … I think they’re used to just going on ward rounds and being more kind of ‘have you done this?’, ‘can you do this?’…you kind of realise it more watching that …”*
b	VREG8	***David*** *: “I think in terms of decision*-*making I think it is (3.0) I feel most of what I do is making decisions … you make decisions based on you know the information you’re given … around kind of discharge issues, CPR [cardio*-*pulmonary resuscitation] issues, you, you are usually expected to (2.0) make the final decision. But often … you’re, you’re offered the decision to make … like CPR decisions the nurses will initiate that and if they ever come to me and say ‘do you think this patient should be for CPR … they’ll come to me in the expectation that I’ll write ‘do not attempt resuscitation’ … yeah, I make the decision, but many times the decision is made and I kind of rubber stamp the decision because that’s how the hierarchy works …”*
c	VREG9	***Specialty trainee Katie*** *: “… in David’s ward round he is the clear leader in that he sees the patients and makes a decision … I’m not saying David is paternalistic because it’s not like that but …* [referring to Fig. 1 clip] *kind of writing over the trolley, speaking to the FY1 writing again, moving away so he can see the FY1’s writing … they* [consultants] *just have so much to do and I think that the way the hospital works is that they do board rounds on the other side of the hospital at 9 o’clock and have patients in other wards … and then their clinics…”*
d	VREG10	***Foundation trainee Douglas*** *: “…it’s mainly just making sure that the paperwork is up to date … it’s just making sure that all the information for the ward round is there for the consultant … it’s our job as junior doctors to try and carry out those jobs …”*
e	VREG3	***Practice nurse Fiona*** *: “… and I prob*- *(laughs while talking) I was probably bossing him about there (laughs) wasn’t I?* *Pharmacist Amy: (Fiona laughing) that’s because it was his first time, you were making sure he knows what he was doing (laughs)* *Fiona: I felt very bossy there (laughing) …* *Primary author: So did you feel like you were leading that, that session?* *Fiona: No (shakes head vigorously) no, no (laughs and looks at Christina the GP who is also present in the video*-*reflexivity session) not by any means …”*
f	VREG5	***GP partner Jason*** *: “… so, from a leadership point of view … it’s about knowledge transfer and at the start of each interaction … [Fiona]’s got the information (1.0) ehm and (3.0) and then (1.0) it’s just a (1.0) discussion I don’t think there’s any kind of (2.0) significant hierarchy (1.0) although I suppose from an overall decision*-*making process (2.0) ultimately I suppose the call is (1.0) is mine … I’m pretty sure she would disagree if she thought I was talking rubbish (laughs) …”*
g	VREG5	***Jason*** *: “… it tends to be a bit of a (3.0.) [moves both hands together in a swinging motion] a swing weighted interaction for each patient because … If there is something that needs to be modified or discussed again sometimes [Fiona]’s quite happy to take it. She’s been doing it for (2.0), ehm, a good while …”*

### Processes of leadership observed in context

Across the data, we most commonly identified clinical leadership influential acts of organising (IAOs), that is, leadership interactions where the focus of the IAOs were decisions about ongoing patient care. Other IAO types included: educational leadership, where the IAO occurred during an educational activity; administrative leadership, for example, decisions about bed management; and change leadership.

We derived from the data common features of an IAO to include some or all of the following processes: (1) information exchange, typically found across all leadership interactions and used to facilitate an IAO; (2) leadership non-negotiation (where there was a clear-cut professional or interprofessional hierarchy) or leadership negotiation, in which it was observed that team members “granted” leadership to the most appropriate member; such granting occurring either overtly through direct designation of leadership or covertly through questioning or non-verbal interactions; and (4) discussion and agreement of a plan (following information exchange and leadership negotiation) or passive compliance of a plan, without offer of opinion, planning or discussion (again related to professional and interprofessional hierarchies).

IAOs of all types were generally led by trained medical staff (i.e. GPs and consultants), in particular during formal contexts such as ward rounds and multidisciplinary meetings. For example, in Fig. 1 we see information exchange between David and Douglas as part of a ward round in which they look at paperwork, with little verbal interaction (see turn 1). There is no negotiation of leadership as David assumes the role of leader throughout and Douglas appears to passively comply with the plan for each patient (see turns 2, 5 and 8).

In contrast, our analysis also revealed IAOs in informal contexts and IAOs where leadership was shared by more than one member of the multidisciplinary team. These interactions were characterised by leadership appearing to be negotiated and re-negotiated throughout an interaction. Thus, leadership could be described within these scenarios as emergent as it happened in immediate response to unpredictable situations. Across the video-observational data, this *leadership emergence* included staff outside traditional professional and interprofessional hierarchies, undertaking shared leadership. For example, in Fig. 2 the interaction between ‘Fiona’ and ‘Jason’ moves through several stages. Initially, there is an exchange of information between the two professionals about the patient (see turns 1 and 2), followed by a period of leadership negotiation (see turns 2–9). At this point, leadership and its associated influences appear to be shared, moving back and forth between the two. Following further information exchange, a plan is discussed and agreed by both parties (see turns 10–12).

### Interactional analysis of processes

Exploring human–human and human–material interactions during these IAOs allowed us to examine in greater detail how leadership was enacted. These included non-verbal interactions; control and/or use of material artefacts; and the use of language.

#### Non-verbal interactions

Physical positioning and eye contact was an important part of the formal leadership interaction between David and Douglas (see Fig. 1, still 1). We see both standing side-by-side facing the patient notes trolley looking through paperwork. This position emphasises a focus on paperwork and seems to discourage eye contact. In the second still in Fig. 1, we see David leaning over ‘checking’ what Douglas is writing, something observed regularly throughout this interaction. Such non-verbal interaction serves to co-construct the hierarchical leadership relationship between this consultant and his trainee.

Less clear-cut is the physical positioning between Jason and Fiona in their interaction. In Fig. 2, still 3 we see Jason sitting at the consultation desk with his back to the camera facing a computer screen and keyboard. Fiona is sitting on a chair (the lower patient’s chair) to the right of the desk (partially facing the camera and partially facing Jason) clutching paperwork, which she holds onto and refers to throughout the interaction. This, on its own, could be interpreted as a visual representation of a traditional hierarchical leadership relationship (with the doctor as leader in the higher chair at the desk; and the nurse as follower in the lower consulting chair). However, bringing together the multiple aspects of our analysis reveals a more complex shared leadership process between Jason and Fiona (as will become clearer later in these findings).

#### Control/use of artefacts

Control and/or use of artefacts was an important factor in leadership IAOs across our data. Patient data in various forms was a key human–material interaction across the footage. Control of materials like, for example, who had sight of patient and nursing notes; computer screens and the keyboard; and who was deciding which patient to discuss on the hospital ward’s white board were key strategies in the negotiation of leadership. Those that were in ‘control’ of these artefacts were the participants who tended to be in the position of leader within the interaction.

For example, in Fig. 1 David not only writes his decisions in the notes (turn 1) but also tells Douglas what to write in the notes that he is working on (see turn 2). Additionally, as mentioned earlier, later in the interaction David moves over to Douglas to check what he is writing in the notes. Thus, David controls the material artefacts (of pen and notes) and this serves to help maintain David’s leadership position throughout the IAO.

In contrast, in Fig. 2, aligning with the notion that the interaction between Jason and Fiona illustrates shared leadership, at the beginning of the interaction Fiona is selecting and providing detail about the patient from her notes, focussing on the patient’s blood pressure. Within this interaction, Jason has no access to the information on these notes and thus Fiona controls the situation through her choice of what information to share. In turn 2, once Fiona has supplied the information and during a pause in talk she lifts her eyes from the notes and leans forward to look at the computer screen to explore the information that Jason has via the computer (see Fig. 2, still 4). Thus, Fiona gains access to all of the information available, in contrast to Jason who only has what is available on the computer screen (so, only partial control of information).

#### Use of language

Directives were typical across the data and implied leadership, particularly in more formal interactions where leadership was less ambiguous. For example, in Fig. 1, there are several instances where David provides directives to Douglas (see turns 2, 3, 5 and 8). These directives are delivered frequently and are expressed as explicit imperatives, for example, “just say she’s uhm, doing well” (turn 2) or “can you just check” (turn 3). There is little explanation for each directive, which may affect Douglas’ understanding of David’s reasoning for the directives. Additionally, direct questioning aimed at obtaining information, and therefore facilitating the IAO is used by David (for example, see turn 1).

In contrast, whilst directives are not used throughout the interaction between Jason and Fiona, questioning was used as a way of carefully negotiating professional boundaries and leadership identities. In Fig. 2, we see questions within this IAO used strategically to do a number of things. For example, in turn 2, Fiona begins her question with “I don’t know whether…” This opener is used as a hedge, which could indicate an awareness of professional boundaries and soften what could be a face threatening act for Jason, as pressure is placed on him by Fiona to influence the management of the patient in a particular direction. At the same time, Fiona establishes her own leadership identity, knowledge and experience of the patient’s condition through indication of the direction that she thinks this discussion should take (in this instance, a review of the drug this patient is taking). Jason responds to Fiona’s question by providing drug information, stating that the patient “…has scope to play with that,” in reference to the information he has on the screen (see turn 3). By stating his opinion, Jason is negotiating his own influence and reaffirming his own leadership identity as the one who can make decisions about this patient’s drugs. He follows this statement up by asking Fiona what other drugs this patient is on: “that aren’t helping” (see turn 3). Although this is a more direct question in which Jason requests information, he is also seeking Fiona’s opinion by asking her what drugs she thinks are sub-optimal. This could mark professional courtesy and respect for Fiona’s knowledge and experience. Fiona responds with a further question (see turn 4) for which they seek out the answer together on the computer screen before she asks once again about the patient’s glycoside (see turn 7). Thus, through questioning, Fiona carefully negotiates her influence in the process and the direction of the discussion without challenging her colleague’s professional identity as traditional hierarchical leader.

Finally, personal pronouns and the words collocated with pronouns were also a way in which leadership identities and relationships were negotiated. For example, in Fig. 1, David tends to use “I” collocated with ‘doing words’ such as “I’m going to” (turn 1) or “I’ll ask” (turn 8), which could indicate his authority in this situation.

In Fig. 2, Jason uses the pronoun ‘I’ in different ways. Firstly, he collocates ‘I’ with verbs such as ‘I would’ (turn 8), and ‘I’ll’ (turn 12) which could indicate his own agency and desire for authority in the decision-making for this patient. Second, and conversely, Jason also collocates ‘I’ with ‘thinking’ verbs such as ‘I mean’(turn 8), ‘I reckon’ (turn 8) and ‘I think’ (turn 12), which could indicate a wish to soften his position and any potential face threatening acts for Fiona, given her clinical knowledge and long-standing experience.

### The VRE groups: influences on leadership processes

When the interaction in Fig. 1 was discussed inVRE group 8, David identified that he held the position of leader (see Table 4: quote (a)). He described his relationship with Douglas as ‘unambiguous,’ emphasising a clear hierarchical relationship between the two. David continues to explore reasons why this leadership relationship was so clear, stating that the frequent rotation of foundation trainees influences their opportunities for more active involvement in the planning of patient care. David also discussed how this affects his approach to leadership, stating it was ‘very much me directing him’ and how watching it on the video emphasised this (Table 4: quote (a)). David accepted that leadership in this context was very much part of his responsibilities as consultant (Table 4: quote (a)). Notable was David’s repeated use of ‘them’ when referring to junior trainees and ‘us’ referring to consultants, serving to create an impression of an adversarial relationship, which further emphasised the hierarchy.

David also emphasised the importance of other interprofessional team members’ roles in clinical leadership IAOs. Table 4, quote (b) depicts the discussion between himself and the ward physiotherapist on the subject. David talked about decisions being ‘offered’ to him to ‘rubberstamp’ because ‘that’s how the hierarchy works’. Thus systems and protocols help to construct David’s formal leadership position.

When this interaction was discussed in VRE group 9 with a higher stage specialty trainee (‘Katie’), she also recognised the clear role of consultants as leaders and the different ways in which their leadership was enacted. She also noted physical positioning (Table 4: quote (c)). This identification of individual behaviours was a common theme discussed across the VRE groups by nurses and allied health professionals. Within his VRE group 10, Douglas also emphasised his own position as junior in this leadership relationship. He described his role within this work environment as very much task-focussed (Table 4: quote (d)). Also commonly discussed within the VRE groups in relation to this clip was time pressure placed on consultants by issues off the ward, with time pressure seen to influence the need to be directive during the ward round and maintain pace.

When the Fig. 2 excerpt was discussed within the VRE groups in Site A, in contrast to our video-observation-based analysis of shared leadership above, the practice nurse Fiona was adamant that her role did not involve leadership. However, she regularly contradicted herself by also suggesting that she was ‘bossing’ Jason (Table 4: quote (e)). Through this discussion within VRE group 3 Fiona perceives a static leadership relationship based on a traditional interprofessional hierarchy. Through her steadfast denial of her own leadership, Fiona expresses her discomfort at the consideration that leadership may be shared.

In VRE group 5, similar to the practice nurse, Jason also seemed verbally clear of his position in the interprofessional hierarchy (Table 4: quote (f)). Although Jason clearly respects the practice nurse’s professional knowledge he also states that the call is his, thus identifying his role as clinical leader. Jason further acknowledges the value of information exchange with his experienced nurse colleague as he describes the planning phase of the process (Table 4: quote (g)). Jason describes the interaction as ‘swing weighted’, suggesting that he is aware of the complexity of the interaction and that the boundaries between leadership relationships are more blurred than a static hierarchical relationship would suggest.

## Discussion

Through this analysis, we have explored at a micro-level how leadership can be enacted in healthcare workplaces. These data show a large number of ‘influential acts of organising’ (IAOs). Our research suggests that leadership in healthcare is enacted within complex adaptive systems (CASs), something that is often discussed within the healthcare literature but seldom researched (Pslek and Greenhalgh 2001; Kernick 2002; Weberg 2012). Within these CASs, many routine issues, such as those captured by the interaction between David and Douglas (Fig. 1), were resolved through traditional hierarchies (Parks 2005). However, we also recorded IAOs, as illustrated by the interaction between Jason and Fiona (Fig. 2), in which leadership was emergent and came from outside traditional interprofessional hierarchies (Uhl-Bien et al. 2008; Lichtenstein and Plowman 2009). These IAOs were negotiated through social interaction between designated or emergent leaders and followers acting within context (Fairhurst and Uhl-Bien 2012).

We were also able to identify some of the typical interactional features that occurred within IAOs. We found leadership to be enacted through human–human interaction and human–material interaction. By analysing these visual aspects, we offer a new perspective on how leadership is constructed in interaction. In other healthcare educational research at the undergraduate level, similar interactional strategies have been found to construct doctor–medical student–patient relationships (Rees and Monrouxe 2010; Rees et al. 2013). Our work, however, is the first to explore these strategies in the context of interprofessional leadership within the professional workplace.

Another original aspect of our research was our inclusion of the VRE groups, which revealed participants’ viewpoints on leadership practices and relationships. Similar to previous research, some discussion within the VRE groups revealed deeply entrenched values, beliefs and practices in relation to healthcare leadership as hierarchy (Linguard et al. 2012; Chriem et al. 2013). This was made overt through the processes of participants viewing themselves in practice during VRE groups. These traditional views of leadership may serve to inhibit the cultural shift towards distributed patterns of leadership inherent in complex adaptive systems.

From a complexity leadership theoretical perspective, identifiable within the interactional data were different integrated forms of leadership (macro, meso and micro) and through our analysis we identified how each level interacts and influences leadership at a micro-level. Leadership was enacted through the local policies and procedures present within each site, through paperwork and IT systems to which participants continuously interacted within the video-observational footage, linking the micro-level system to those at the meso-level (for example, between wards) and macro-level (for example, organisation-wide systems).

### Methodological strengths and limitations

The use of VRE allowed for engagement with leadership complexity, enabling us to explore the whole workplace as a dynamic learning system. It allowed us to visibilise practice in the moment rather than discussing general leadership behaviours that were simplified or reassembled through field notes and memory (Iedema et al. 2007; Mesman 2007; Carroll et al. 2008; Linguard et al. 2012; Chriem et al. 2013). Using video, we could look at the minutiae of interactions (i.e. micro-level interactions) within each of the two workplaces studied. For example, we could identify the embodiment of leadership through the control and use of artefacts. Previous observational studies of leadership in healthcare have not used video to record such leadership interactions (Linguard et al. 2012; Chriem et al. 2013).

Despite this, we were also mindful of the extent to which participants would actively construct the interactions captured, even though they appeared to be ignoring the camera and the primary author’s presence (Lomax and Casey 1998). Different participants afforded different prominence to the experience of being videoed, thus the process was at all times fluid and unplanned (Forsyth 2009). We believe that the video-camera became more than a mere recording device, but was a presence in the research in its own right, allowing participants to generate their own understandings about leadership processes within their workplaces (Forsyth 2009).

Viewing the video-footage of their everyday workplace activities within the reflexivity sessions provided opportunity for participants not only to re-experience the complexities of their healthcare workplace, but also to view those complexities from a new angle (Iedema et al. 2013). Through this, participants were ‘confronted by the deeply familiar in a way that rendered it strange’ (Iedema et al. 2013: p. 8). Despite editing and compressing multiple interactions in time into short edited clips, a small piece of footage would elicit long discussion within the video-reflexivity sessions and lively description of participants’working lives (Iedema et al. 2013). Thus, participants would ‘fill in the gaps’ through their own experiences and familiarity of the contexts (Carroll 2009). These multiple realities were co-constructed in discussion with the primary author, who had spent time within their workplace contexts and had become familiar with the daily routines and participants’ roles within them (Carroll 2009; Iedema et al. 2013).

It is important to note, however, that during the process of editing, the research team had to make decisions about what to include, which would have influenced what was discussed in the reflexivity sessions and ultimately how participants constructed their healthcare leadership experiences. Also noteworthy is that participants made careful choices of words during the reflexivity sessions, possibly reflecting who was listening (for example, Fiona’s reflexivity session included another GP and Jason’s reflexivity session included other senior nurses). Finally, using video-reflexivity provided formal accountability for our own analysis and provided rich opportunity for the co-construction of meaning which served to enrich our study findings (Iedema et al. 2007; Carroll et al. 2008).

#### Educational implications

This research highlights both professional hierarchies and the complex emergence of leadership in the healthcare workplace. Thus, we argue that our research emphasises the need for leadership education at all levels including both intra- and interprofessional activity. Current healthcare literature asserts the need to shift to distributed patterns of leadership that are adaptive to complex healthcare systems (Royal College of Physicians Canada 2013; NHS Leadership Academy 2013; West et al. 2015). Focusing on developing learners’ understandings of the systems in which leadership emerges would help students and trainees to move away from the notion that leadership is only about position within a hierarchy. Additionally, providing interprofessional leadership education that is contextually relevant will encourage open discussion about the interplay between professional boundaries and changing leadership processes, which may help to dismantle traditional interprofessional hierarchies. In order to do this, leadership education situated in the workplace with the whole interprofessional team is imperative.

We particularly suggest that VRE, in addition to being used as a research methodology, could be employed within leadership education. By viewing themselves in practice, participants could explore opportunities for change and improvement in leadership practices through open discussion about their everyday but often unseen work. VRE could help develop the ability of teams to lead their way out of problems through reflexive discussion.

#### Implications for future research

Our study explores one snapshot in time with respect to workplace-based leadership practices. Longitudinal research is now key to provide insights into how healthcare leadership develops over time. Moreover, longitudinal work would allow additional exploration of how macro-, meso- and micro-systems interrelate with different leadership activities that are enacted. Repeated VRE ‘cycles’ or a participatory action research approach would help to address this (Bleakley and Cleland 2015). Additionally, whilst it was not within the scope of this paper to undertake analysis of group dynamics within the VRE groups, we would suggest that this would make for interesting future research, using different lenses to explore issues such as power, negotiation and responsibility. Finally, any future research should focus on leadership as a process (taking in individuals, relationships, contexts and systems), rather than individual leaders.
